# High-content analysis of tumour cell invasion in three-dimensional spheroid assays

**DOI:** 10.18632/oncoscience.171

**Published:** 2015-06-14

**Authors:** Vinton Cheng, Filomena Esteves, Aruna Chakrabarty, Julia Cockle, Susan Short, Anke Brüning-Richardson

**Affiliations:** ^1^ Leeds Institute of Cancer and Pathology, University of Leeds, UK; ^2^ St. James's University Hospital, Leeds Teaching Hospitals NHS Trust, UK

**Keywords:** Glioma, spheroids, invasion, 3D immunohistochemistry

## Abstract

Targeting infiltrating tumour cells is an attractive way of combating cancer invasion and metastasis. Here we describe a novel and reproducible method for high content analysis of invading cells using multicellular tumour spheroid assays in a high grade glioma model. Live cell imaging of spheroids generated from glioma cell lines, U87 and U251, gave insight into migration dynamics and cell morphology in response to anti-migratory drugs. Immunofluorescence imaging confirmed cytoskeletal rearrangements in the treated cells indicating a direct effect on cell morphology. Effect on migration was determined by a Migration Index (MI) from brightfield images which confirmed anti-migratory activity of the drugs. A marked effect on the core with treatment suggestive of disordered proliferation was also observed. A newly developed technique to prepare the spheroids and migratory cells for immunohistochemistry allowed an assessment of response to drug treatment with a selection of markers. A difference in protein expression was noted between cells maintained within the core and migratory cells indicative of the presence of cell subpopulations within the spheroid core. We conclude that this high content analysis allows researchers to perform screening of anti-tumour invasion compounds and study their effects on cellular dynamics, particularly in relation to protein expression, for the first time.

## INTRODUCTION

The targeting of invading cells is a novel approach to control the dissemination and growth of high grade gliomas (HGGs) [1]. Tumour cell invasion is regulated by a number of complex signalling pathways but the contributing cell-cell and cell-matrix interactions remain poorly understood [2]. It has been proposed that invasiveness is related to stem cell-like qualities of a distinct subpopulation of glioma cells [3]. Elucidating the phenotypic differences between invading and non-invading glioma cells will allow the development of agents specifically targeting this phenomenon.

Multicellular tumour spheroids (MCTS) are formed *in vitro* through small cell aggregates. They are increasingly being used in cancer research to simulate the three-dimensional (3D) organisation of an *in vivo* tumour mass and thus replicate the tumour microenvironment in a simplified model [4]. When embedded into a hydrogel matrix, for example collagen or Matrigel, individual invading cells can be monitored as they migrate out from the 3D structure using time lapse video microscopy [5]. Immunohistochemistry (IHC) represents a complementary way of studying the expression of specific antigens in MCTS [6]. However, the ability to perform large-scale analysis of invasive migratory cells in a reproducible fashion with MCTS has proven to be challenging due to issues such as matrix variability, difficulty controlling spheroid formation and a lack of high-resolution methodologies that allowed invasive cells to be identified and phenotypically interrogated. Here, we aimed to assess the feasibility of demonstrating the effect of anti-migratory drugs and the presence of cell subpopulations for targeting cell invasion in a 96-well based platform for large scale analysis. The anti-migratory activity of two known inhibitors, Lithium chloride (LiCl) and Bio-Indirubin (BIO), was investigated as an example in a MCTS glioma 3D model utilising a range of investigative technologies. We have developed a novel approach that combines a workflow strategy with protein expression analysis for studying 3D HGG cell invasion in a 96-well based assay, permitting the identification of specific biomarkers in this population subset.

## RESULTS

### 96-well based analysis by live cell imaging

**Figure 1:**
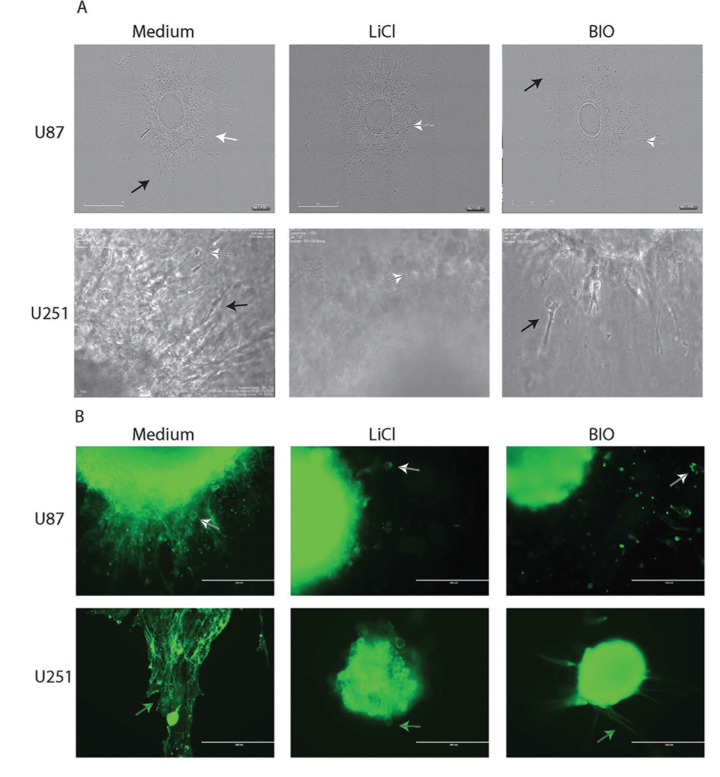
The effect of drug treatment on migration dynamics **(A)** Endpoint stills of U87 and U251 spheroids embedded in collagen after no treatment or treatment with LiCl or BIO. Elongated migratory cells are observed further away from the original core (black arrow) whilst rounded cells are associated with the core (white arrow). Treatment with LiCl leads to a more rounded phenotype (white arrow) whereas treatment with BIO is characterised by a mixed population of both rounded (white arrow) and elongated cells (black arrow). Please note that cores for U251 are shown at a higher magnification as the original cores were smaller than those generated for U87. Scale bar = U87: 900 μM: U251 10 μM. **(B)** Spheroids prepared for immunofluorescence after treatment. IF labelling confirms the effects of drug treatment observed by live cell imaging. Cytoskeletal rearrangements are seen in U87 and U251 after the addition of LiCl and BIO. In U87 actin labelling is more pronounced on the cell cortex of migratory cells (white arrow) while in U251 actin stress fibres in untreated cells appear to be reduced in treated cells (green arrow). Rounded morphologies are more abundant in both cell lines after treatment with LiCl. Scale bar = 200 μM.

A workflow was devised for analysing collagen-embedded glioma spheroids maintained in a 96-well plate using live cell imaging, immunofluorescence and immunohistochemistry (IHC). MCTS were formed in an ultra-low adherence (ULA) 96-well plate from established HGG cell lines plated 72 hours earlier. These MCTS were embedded in an extracellular matrix composed of type I rat tail collagen. Compared with the hanging drop method of producing uniform spheroids, this method requires minimal handling and each well permits individual assay of a drug treatment [4]. Our tumour spheroids were treated with glycogen synthase kinase-3 β (GSK-3β) inhibitors BIO and LiCl, which have previously been shown to specifically inhibit tumour cell invasion [7–9]. Multicellular spheroids were generated from established glioma cell lines U87 and U251, embedded in collagen and treated with the two known GSK-3 β inhibitors. Using this platform the effect of drug treatment was first assessed by live cell imaging. From these data we were able to recognise distinct migratory behaviour. When close to the spheroid edge migratory cells maintained a more rounded morphology that changed into an elongated morphology the further away the migratory cells travelled. There appeared to be a constant “halo” of migratory cells close to the core edge migrating in a directional fashion. This was observed for both cell lines, however, in U251 migratory cells appeared to migrate in a chain-like fashion away from the core giving them the appearance of spikes emanating from the core. When treated with LiCl the number of migratory cells appeared to be decreased and the “halo” thinned out. Morphologically more cells appeared rounded even when further away from the core. BIO treatment again led to a reduction in the number of migratory cells in both cell lines; treatment resulted in a population consisting of both rounded and elongated cells in the case of U87. U251 treated spheroids were characterised by migratory cells that appeared stretched and blebby and not able to detach from the original core (Figure 1A).

### Immunofluorescence analysis of cell invasion into collagen

To confirm the phenotypes observed by live cell imaging we established a method to stain the untreated and treated spheroids and migratory cells whilst still maintained within the collagen matrix. This allowed the analysis of the cytoskeletal structure of migratory cells for U87 and U251. In untreated U87 cells we observed a migratory cell population consisting of rounded and elongated cells with diffuse actin labelling which was mainly associated with the cell cortex. U87 spheroids treated with LiCl appeared to result in mainly rounded cells, whereas treatment with BIO led to a mixed population of rounded and elongated big migratory cells with pronounced actin labelling. U251 spheroids appeared to produce elongated migratory cells in chains with pronounced actin stress fibre labelling. This disappeared in response to drug treatment, where the LiCl-treated cells had a distinct rounded morphology with no actin stress fibres and the BIO-treated migratory cells appeared as elongated thinner cells with no apparent actin stress fibres (Figure 1B). Thus, live cell imaging and immunofluorescence enabled us to gain first insight into invasion dynamics and cytoskeletal morphology within a 3D setting over a treatment time course. These results demonstrated that treatment with GSK-3β inhibitors resulted in significantly reduced matrix invasion and cytoskeletal rearrangements, particularly affecting actin organisation and focal adhesions (unpublished data).

### Effect of drug treatment on cell migration and spheroid core size by brightfield microscopy

**Figure 2:**
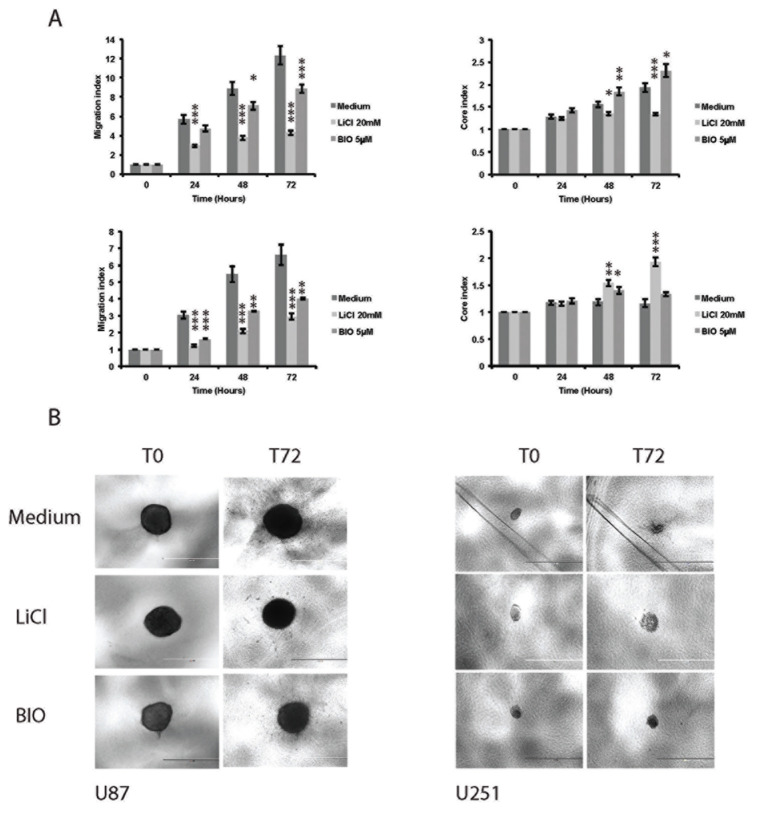
The migratory abilities of U87 and U251 and spheroid core sizes are affected by treatment with the GSK-3 β inhibitors **(A)** The effect of the two inhibitors LiCl and BIO on the MI and core size in U87 (top) and U251 (bottom). At 72 hours BIO had the most inhibitory effect on cell migration wheras LiCl appeared to negatively affect core proliferation. Data are presented as mean +/− SEM. MI T24: LiCl, p < 0.0001; BIO, p < 0.0001; MI T48: LiCl, p < 0.0001; BIO, p = 0.023; MI T72: LiCl, p < 0.0001; BIO, p < 0.0001. Core size change T24 not significant; T48: LiCl, p = 0.047; BIO, p = 0.008; T72: LiCl, p < 0.0001; BIO, p = 0.26. In U251 LiCl showed the most anti-migratory activity at 72 hours but appeared to enhance core proliferation. MI T24: LiCl, p < 0.0001; BIO, p < 0.0001; MI T48: LiCl, p < 0.0001; BIO, p = 0.001; MI T72: LiCl, p < 0.0001; BIO, p = 0.001. Core size change T24 not significant; T48: LiCl, p = 0.01; BIO, p = 0.049; T72: LiCl, p < 0.0001; BIO, not significant. **(B)** Stills of invasion assays at T0 and T72. In both U87 and U251, migration is greatly reduced and the effect on core size is visible by eye after treatment with LiCl. Scale bar = 1000 μM.

Quantification of migration using MI demonstrated that both LiCl and BIO produced a noticeable reduction in invasive capacity compared to control spheroids (Figure 2A and B). In both cell lines LiCl had the more profound effect on migration with a decrease in more than half that observed in the medium only-incubated spheroids. BIO also produced a similar effect, but generally less pronounced at all time points. Observed changes in spheroid core size and nuclear morphology suggested that proliferative activity and cell death were also affected (Figure 2A and B). We noted a distinct differential response in the size of the MCTS core dependent on invasion inhibition at 24 hour intervals compared to at time zero. Interestingly, in U87 spheroids the core size incrementally increased with BIO-incubation and no treatment, although BIO-treated spheroids expanded slightly more at each time point. Conversely the LiCl-treated MCTS initially expanded, but then core size plateaued for the remaining observation period. With U251 spheroids the contrast in core size changes between the treatment groups was more striking. Here we observed that the LiCl-treated MCTS core increased greatly in size at each time point, expanding to nearly twice its baseline area. Surprisingly however the control and BIO-treated spheroids showed a reduction in core size towards the later timepoints (Figure 2A). Statistical analysis confirmed that from 48 hours (T48) the differences in MI between the control group and treatment groups were significantly different for both cell lines. For the effect on core size at 48 and 72 hours, the differences were significant in U87 and at 48 hours for all treatments in U251. However, at 72 hours, only the result obtained for LiCl was significantly different to the control.

### Immunohistochemistry of collagen embedded core maintained cells and migratory glioma cells

**Figure 3:**
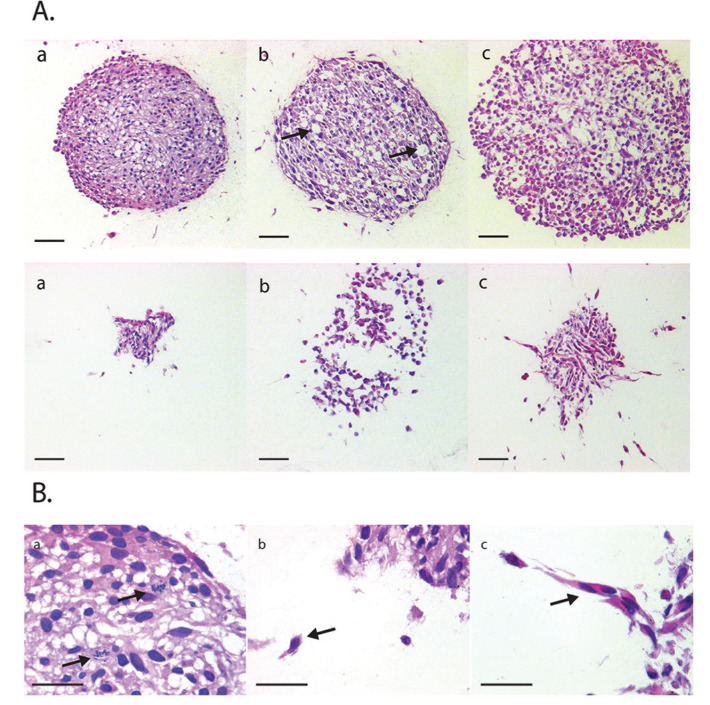
H&E stained sections of FFPE spheroids treated with anti-invasion agents **(A)** *Top row*: U87 spheroids, untreated (a), 20 mM LiCl (b) and 5μM BIO (c). Scale bar= 100 μM. *Bottom row*: U251, untreated (a), 20mM LiCl (b) and 5 μM BIO (c). Scale bar = 50 μM. **(B)** Representative images of mitotic figures visible in stained spheroid (a) and morphological differences between invading cells in untreated U251 spheroid (b) compared to BIO-treated U251 spheroid (c). x40 magnification. Arrows indicate mitotic features in (a) and migrating cells in (b) and (c).

We wanted to determine the underlying processes that were leading to these observations. Therefore we employed IHC to detect biomarker expression patterns, focusing on the challenge of detecting differences between the cell populations in the main spheroid core and those invading into the collagen matrix. To enable histological analysis of collagen-embedded spheroids we developed a new IHC methodology where collagen-enclosed MCTS and their migratory cells were fixed and embedded upon experimental completion. This method included brief washing steps with PBS and fixation of spheroid core and migratory cells in paraformaldehyde while still contained within the original 96-well plate, followed by processing into a paraffin wax block. Sectioning of these formalin-fixed paraffin embedded (FFPE) specimens demonstrated that the spheroid architecture was maintained within the collagen matrix. Notably, we observed that for the first time using an IHC-based method, individual invading cells could be delineated from the spheroid core. Strikingly, initial examination of morphological characteristics using haematoxylin and eosin (H&E) staining showed that specific differences between the treatment groups and controls could be easily identified. In particular we noted evidence of altered proliferative activity by examining mitotic figures, as well as differences in necrosis, apoptosis and cyst formation within the core. Remarkably, invading cells were also noted to be morphologically different to those in the main spheroid mass in following drug treatment (Figure 3 A and B). Whereas in the LiCl treated migratory cells we observed mainly a rounded morphology, in the BIO treated cells a mixture of elongated and rounded cells was present. These observations were entirely in keeping with our previous analyses of invading cells obtained using phase microscopy, live cell and brightfield imaging and immunofluorescence.

### Protein expression in core maintained and migratory glioma cells by immunohistochemistry

**Figure 4:**
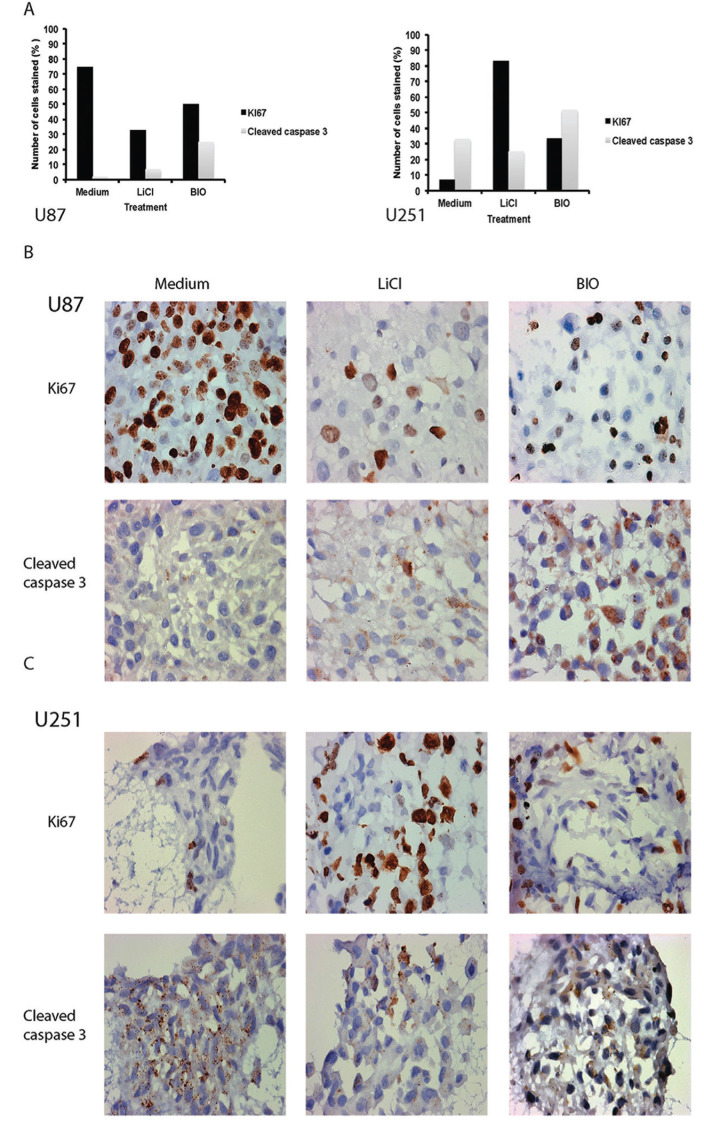
**(A) Graphic representation of staining patterns showing the percentage of U87 (left) and U251 (right) stained for the two markers of proliferation and apoptosis, Ki67 (nuclear stain) and cleaved caspase 3 (cytoplasmic stain)** Expression of Ki67 and cleaved caspase 3 change in response to treatment with the inhibitors LiCl and BIO. **(B)** Representative images for Ki67 and cleaved caspase-3 staining in U87 non-treated versus treated spheroids. x10 magnification. **(C)** Representative images for Ki67 and cleaved caspase-3 staining in U251 non-treated versus treated spheroids. x10 magnification.

**Figure 5:**
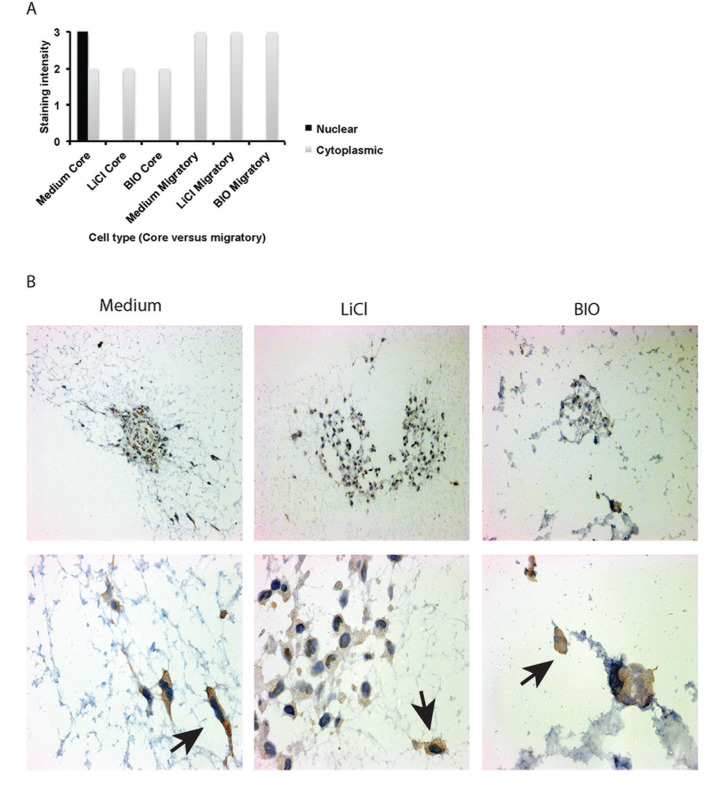
FFPE sections of U251 spheroids displaying differential staining intensities in cells contained within the core and migratory cells after treatment **(A)** Graphic illustration of staining patterns and intensities observed for SOX-2 expression in core maintained cells and migratory cells. **(B)** SOX-2 antigen staining in spheroid core cells and migratory cells after no treatment (Medium), 20 mM LiCl, 5 μM BIO, x10 magnification. Corresponding images at x40 magnification show SOX-2 expression in invading cells and cells maintained within the core with treated specimens (*bottom row*). In treated cells SOX-2 levels are higher in migratory cells than in the cells maintained within the core.

Sectioned FFPE spheroids were then optimised and stained for markers of proliferation (Ki67), apoptosis (cleaved caspase-3) and stemness (SOX-2, nestin) (Supplemental Table 1 – 4). Marked differences in antigen expression could be identified between cell lines as well as between treatment groups (Table 1). As shown in Figure 4, proliferation (Ki67 marker) appeared to be downregulated with inhibition of invasion in U87 spheroids, particularly using LiCl, whilst conversely the opposite effect was seen in U251 spheroids. This proliferation marker expression corresponded inversely to the presence of apoptosis as indicated by cleaved caspase-3 expression. We noted increased cleaved caspase 3 expression in U87 after treatment with LiCl and BIO and also in U251 after treatment with BIO (Figure 4 A-C). These observations appear to confirm our findings from the data on core size over time in the invasion assay that altered proliferative and apoptotic events within the spheroid core occur as a result of treatment with especially LiCl. Stemness markers were ubiquitously present and not greatly altered by inhibiting invasion. Nestin expression appeared to be strong in all cells despite treatment, however, in U251 after BIO-treatment staining intensities appeared decreased in comparison to the other two conditions (Supplemental Figure 1). The proportion of cells stained did not change even with treatment. We also noted changes in SOX-2 expression (Tables 2 and 3). U251 spheroids showed decreased SOX-2 expression within the core when treated with LiCl or BIO (Table 3). Interestingly, highly migratory cells revealed different SOX-2 expression levels compared to cells remaining in the spheroid core. For example, we noted that although SOX-2 was expressed in both cell lines, the expression level only changed in response to treatment in U251 cells. After treatment, expression was decreased in the cells remaining within the core but continued to be highly expressed in the migratory cells (Figure 5, Table 3).

**Table 1:** Results of antigen marker expression based on IHC for MCTS from two different glioma cell lines (U87 and U251) treated with invasion inhibitors

Marker	U87			U251		
	Control	LiCl	BIO	Control	LiCl	BIO
Cleaved caspase-3	+ 1	+ 1	+ 2	+ 2	+ 1	+ 3
Nestin	+ 4	+ 4	+ 4	+ 4	+ 4	+ 4
SOX2	+ 4	+ 4	+ 4	+ 4	+ 2	+ 3

The remaining antigens (cleaved caspase 3, nestin and SOX-2) are discretely grouped into quartiles according to proportion of specimen stained (0=No cells stained; +4=76–100% cells stained)

**Table 2:** Results of antigen marker SOX-2 expression based on IHC for MCTS vs migratory cells from U87 treated with invasion inhibitors

Marker expression	Cells maintained within core			Migratory cells		
	Medium	LiCl	BIO	Medium	LiCl	BIO
% of cells stained	100	100	100	100	42.8	80.7
Staining intensity	3	2	3	3	3	3

% of cells stained were the number of cells labelled with the SOX-2 antibody. Staining intensity was scored as 3 – strong; 2 – medium; 1 – low and 0 – no staining. For both the cells maintained within the core and the migratory cells a pronounced cytoplasmic localisation was observed. We also noted a particularly strongly labelled region of cells on the rim of the core in the untreated MCTS, which disappeared with treatment

**Table 3:** Results of antigen marker SOX-2 expression based on IHC for MCTS vs migratory cells from U251 treated with invasion inhibitors

Marker expression	Cells maintained within the core			Migratory cells		
	Medium	LiCl	BIO	Medium	LiCl	BIO
% cells stained	100 (C)	100 (C)	100 (C)	96.4 (C)	22.2 (C)	100 (C)
	87.7 (N)	0 (N)	0 (N)	0 (N)	0 (N)	0 (N)
Staining intensity	2 (C)	2 (C)	2 (C)	3 (C)	3 (C)	3 (C)
	3 (N)	0 (N)	0 (N)		0 (N)	0 (N)	0 (N)

% of cells stained were the number of cells labelled with the SOX-2 antibody. Staining intensity was scored as 3 – strong; 2 – medium; 1 – low and 0 – no staining. For both the cells maintained within the core and the migratory cells a pronounced cytoplasmic localisation was observed. In addition, we also noted an additional, particularly strongly, labelled nuclear localisation which disappeared with treatment. Cytoplasmic labelling only was seen in treated cells in both the core and among the migratory cells. (C=cytoplasmic, N=Nuclear)

## DISCUSSION

In this study we hoped to achieve high content analysis of cell invasion in three dimensional spheroid assays utilising different technologies. We firstly noted that the generation of spheroids in 96-well low adhesion plate was an efficient means to produce uniform spheroids, which we were then able to analyse for the migratory behaviour of cells emanating away from the original spheroid core and the response to targeting with anti-migratory drugs. Live cell imaging gave us first insight into cell migration dynamics in a 3D setting with differences in the modes of migration of the cells derived from the two cell lines and also changes in morphology in response to drug treatment among the two cell lines. Immunofluorescence analysis after assay completion and MI data also confirmed our first observations highlighting the overall anti-migratory effect of the two inhibitors as well as differences in terms of proliferative activity. IHC allowed the closer analysis of protein expression levels in cells contained within the core and migratory cells. We were interested in the expression of the proliferation marker Ki67 to detect changes in proliferation, the apoptotic marker cleaved caspase-3 for possible apoptotic events and the two stemness markers nestin and SOX-2 to detect stemness-like subpopulations in the core and among the migratory cells. We were able to detect specific changes among the U87 and U251 spheroids and migratory cells; in U87 treatment led to a decrease in proliferation and increase in apoptosis in core maintained cells, whereas in U251 we observed a marked increase in proliferation in response to the two drugs. It is known that both LiCl and BIO target GSK-3 β and recent studies have shown that GSK-3 β regulates differentiation in glioblastoma [10, 11]. LiCl was found to induce tumour cell differentiation and also enhanced apoptosis in glioma cell lines and glioblastomas. We also noted an effect on proliferation and apoptosis, however, there was a difference among the cell lines, which has also been noted in other publications [10] and highlights the heterogeneity of this tumour type and a caveat for drug development. Interestingly, we found some changes in SOX-2 levels, which is a marker of stemness. In especially U251, SOX-2 levels decreased in response to treatment suggesting a drive towards differentiation, but SOX-2 levels remained high in the escaping migratory cells. Recently, several publications have highlighted the pleiotropic role of SOX-2 in GBMs including a critical role for adhesion and migration [11, 12]. Apart from its role in the development of the central nervous system, SOX-2, a high mobility group (HMG)-box containing transcription factor, has also been implicated in oncogenic tumour progression [13]. In some cancers, including breast, colorectal, ovarian and gastric cancers, SOX-2 expression was found to be negatively correlated to patient survival, prognosis and therapy. In other cancers such as hepatocellular carcinoma and lung, NSCLC and squamous cell SOX-2 expression was correlated to improved survival and patient outcome [13,14]. In studies relating to glioma research there is conflicting evidence with regards to SOX-2 and tumorigenicity [15]. Alonso *et al* [16] reported a decreased migratory ability after SOX-2 knockdown in malignant gliomas. In another study by Oppel *et al* [17], the role of SOX-2 in migration was investigated by shRNA in various glioma cell lines. Astonishingly, contrary to their assumption that SOX-2 silencing would lead to decreased migration, an increased dissemination of glioma cells was observed. This was attributed to a switch to a RhoA dependent amoeboid migration characterised by a reorganisation of the actin cytoskeleton and the appearance of membrane blebs [16, 17]. Our own results suggest a role in migration for SOX-2, which appears to be cell line specific. In U87, SOX-2 expression was not affected by drug treatment whereas we noted a decrease in expression in U251 in cells maintained within the spheroid core. U251 migratory cells after treatment with BIO changed their morphology and had higher SOX-2 levels than the cells within the core. The differences between the two cell lines and the effects on migration will be explored further but seem indicative of different survival strategies employed by the two cell types. All in all, using this new technique we now have clear evidence that subpopulations of cells exist within the spheroid cores. For the first time we can now hypothesise that certain properties of these populations are advantageous for GBM survival in response to treatment. We have also identified differences between spheroids generated from the two different cell lines in terms of proliferative activities and the expression of specific proteins, work which was previously not feasible, which highlights the need for a more personalised approach to disease management.

**Figure 6:**
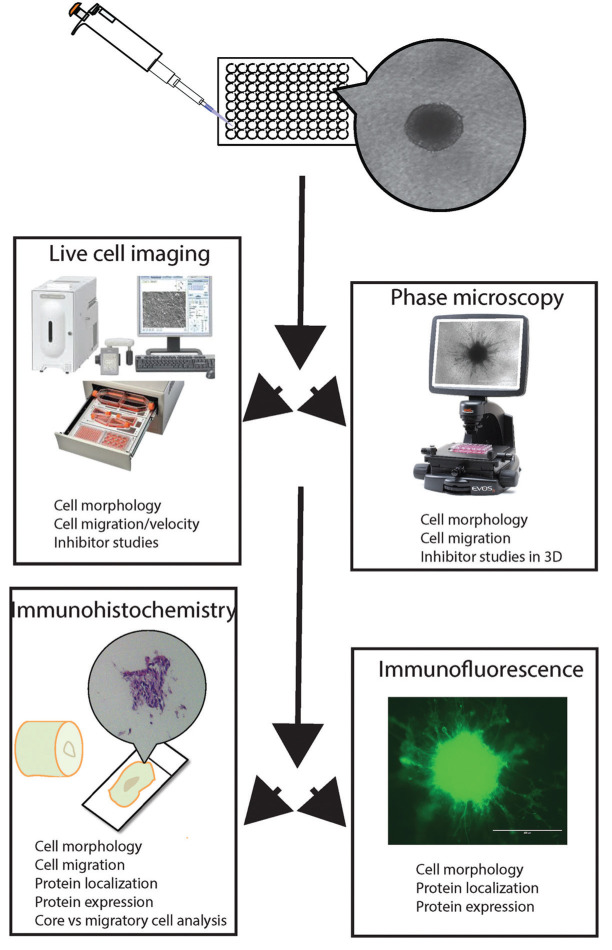
Schematic diagram illustrating the stepwise utility of multicellular glioma spheroids cultured in 96 well ULA plates After 72 hours incubation, the spheroids are embedded in a type I rat tail collagen matrix and treated with invasion inhibitors. They can then be imaged over time using brightfield microscopy to assess the invasive ability of the glioma cells, based on distance travelled from the spheroid core. It is also possible to perform live cell imaging to examine the invasion into the collagen matrix in real time using a BioImager IM or Incucyte System. Upon completion of invasion monitoring at 72 hours, the collagen-embedded spheroids are fixed and stained to perform immunohistochemistry and immunofluorescence.

We report a novel, reproducible and powerful method for investigating the invasive behaviour of cells in a 3D experimental model that allows high throughput screening and high content analysis. We confirmed cytoskeletal rearrangements and changes in migration dynamics in response to drug treatment. In addition, using this we have shown for the first time that distinct subpopulations of cells exist in the spheroids generated in this model, enabling some to escape from spheroids under unfavourable conditions. This new method can be used alongside quantitative molecular techniques such as mass spectrometry and Western blotting to determine the proteomic profile of invading tumour cells [18,19] (Figure 6). It provides an additional tool for researchers to quantitatively cross-validate potential biomarkers in a unique population subset, which has previously proven challenging to characterise, using a multitude of techniques. We are now in the process of separating escaping single cells from the main cell body for detailed analysis. We have also used this system on other cell types, for example paediatric HGGs and epithelial derived tumour cells such as ovarian cancer cells and were able to replicate our results. This new methodology should also be adaptable to automated imaging and analysis workflows, a possibility that we are exploring in our in-house screening facility which uses a PerkinElmer Operetta high content/high throughput imaging system that, when coupled with PerkinElmer Columbus image analysis software, can be programmed to quantitatively examine a specific visual phenotype. If successful this would allow the use of our new technique in higher-throughput applications while also removing any influence of observer bias from the analysis process, further extending the applicability and power of our new methodology.

## MATERIALS AND METHODS

### Spheroid formation

Established cell lines for adult HGG (U87MG and U251) were cultured to 70% confluence. Cells were seeded at a 0.5 × 10^5^ cells/ml density in 96-well ultra-low adherence plates (Costar). Over the course of 72 hours these cells were induced to aggregate into a multicellular spheroid with an estimated density of 5,000 cells. Once MCTS were confirmed as fully formed under light microscopy, they were embedded in rat tail type I collagen (Corning) within each well.

### Invasion inhibition assay

Inhibitors of glioma invasion - lithium chloride (LiCl, Sigma) and Bio-Indirubin (BIO, Calbiochem) - were used to investigate effects on cell migration dynamics and protein expression in invading cells, and non-invading cells within the spheroid core. The spheroid within each well was incubated in a compound mixture at concentrations known to inhibit invasion (LiCl 20 mM; BIO 5 μM) over 72 hours. Live cell imaging over 48 hours using a Nikon BioStation IM or the IncuCyte ZOOM system (Essen BioScience) and time-lapse imaging EVOS inverted microscope (AMG) at 24 hour time points were performed to assess extent of cellular invasion into the collagen matrix. Control spheroids received no treatment and were cultured in cell media alone. Each treatment branch had triplicate repeats to check for internal consistency. Images were analysed and Migration Index (MI) for invasion edge was determined as previously described [20].

### Immunofluorescence analysis

After completion of the inhibitor treatment, spheroids and migratory cells whilst still maintained within the collagen matrix were washed three times with PBS and then fixed with 4% paraformaldehyde (PFA) for 30 min at room temperature. They were then permeabilised by the addition of 0.1% Triton-X100 PBS for 10 minutes at room temperature. After another three washes with PBS a blocker was added to the wells (0.05 % Marvel-PBS) and allowed to incubate for 15 minutes. The blocker was replaced with the staining solution containing DAPI and Phalloidin dyes (Life technologies), which were allowed to incubate for another hour. The collagen plugs were washed three times with PBS and then viewed by immunofluorescence microscopy.

### Immunohistochemistry

Following the 72 hour testing period, each collagen-embedded MCTS was washed three times with PBS and fixed in 4% PFA for 24 hours prior to tissue processing and paraffin wax embedding. FFPE specimens were sectioned into 5 μm thin slices through the diameter of the MCTS core. Sections were used for H&E staining, as well as IHC. Immunohistochemical stains were used to measure stemness markers – SOX-2 (R&D Systems), nestin (R&D Systems); proliferation marker – Ki67 (Thermo Scientific); and apoptotic marker – cleaved caspase-3 (Cell Signaling Tech). Antigen retrieval was obtained through a Heat-Induced Epitope Retrieval (HIER) technique with either citrate (pH 6) buffer (Menarini Diagnostics) or EDTA (pH 8) buffer (Menarini Diagnostics) [21]. SOX-2 and nestin were retrieved in the pH 6 buffer, whilst Ki67 and cleaved caspase-3 required the pH 8 buffer. All specimens were incubated for 1 hour in each antibody, with the exception of cleaved caspase-3 requiring an overnight incubation period. Antibody dilutions were prior titrated to reach optimum staining pattern and intensity as follows: SOX-2 (1:50), nestin (1:100), Ki67 (1:100) and cleaved caspase-3 (1:100).

The method for analysing the IHC for each antibody examined both protein localisation and expression levels. We utilised two scoring systems based on either a quantitative or semi-quantitative method. Proliferation was scored based on proportion of Ki67 stained nuclei to unstained nuclei. For the other markers, which stained the cytoplasm or membrane, a semi-quantitative approach was adopted. This involved grouping into quartiles according to proportion of cells stained (0 = no stain; +1 = 1–25% stain; +2 = 26–50% stain; +3 = 51–75% stain; and +4 = 76–100% stain). A secondary scoring system involved semi-quantitative measurement of stain intensity (0 = no stain; +1 = weak; +2 = moderate; +3 = strong). Finally, we performed a qualitative assessment by noting the distribution of stain within the spheroid core and amongst the invading cells.

### Statistical analysis

Statistical analysis was carried out using SPSS and one way ANOVA test. The results were subjected to a post-hoc analysis (Tukey test). Results with a p value of less than 0.05 were considered statistically significant.

## SUPPLEMENTARY MATERIALS FIGURE AND TABLES


