# Supplementary material for: High-content analysis of tumour cell invasion in three-dimensional spheroid assays
Source: Oncoscience. 2015 Jun 14;2(6):596–606. doi: 10.18632/oncoscience.171 (PMC4506363; doi:10.18632/oncoscience.171)
Supplement: Supplementary file 1 [file oncoscience-02-596-s001.pdf]

High-content analysis of tumour cell invasion in three-dimensional spheroid assays

Supplementary Materials

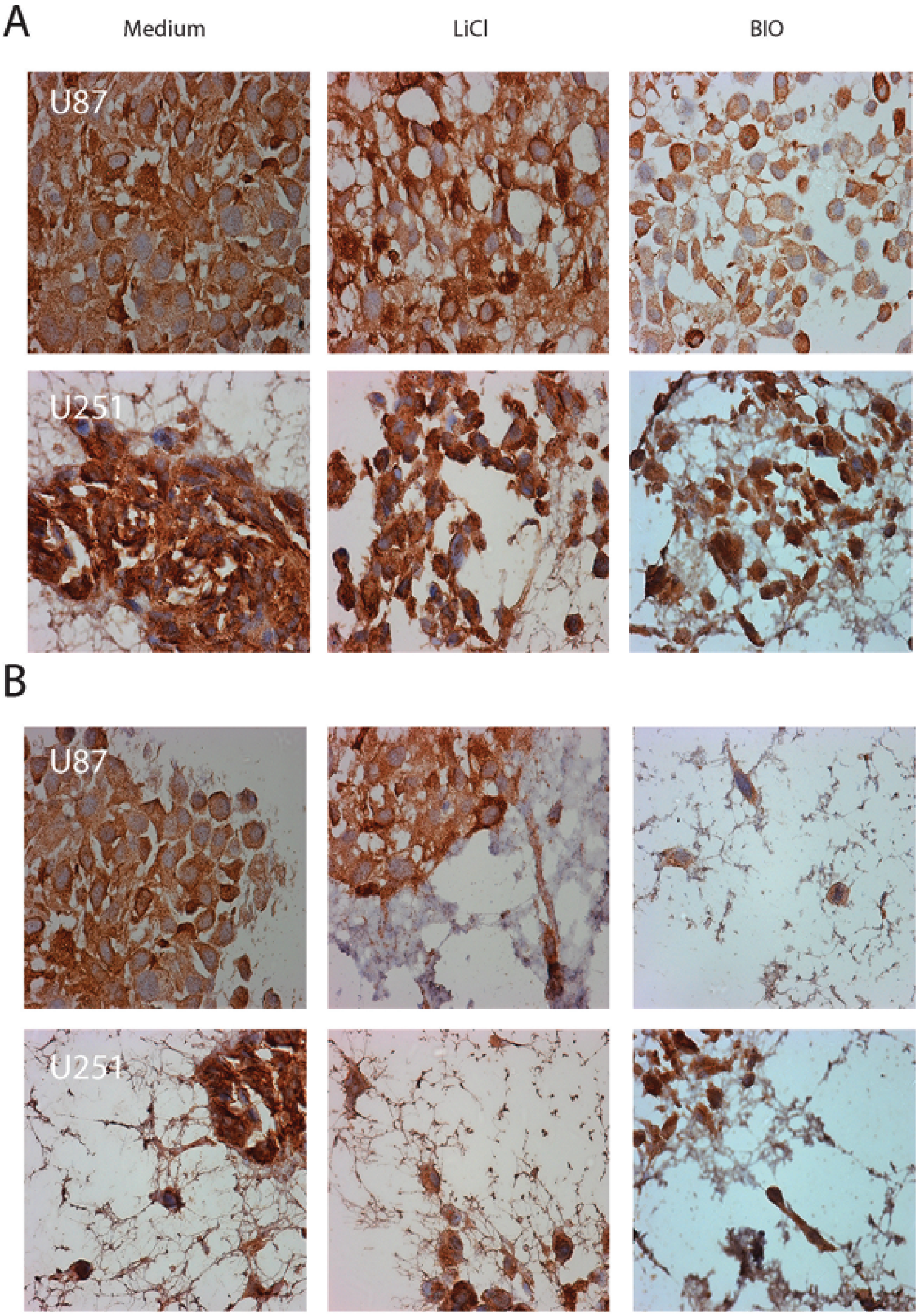

Supplemental Figure 1: Staining patterns observed for nestin in U87 and U251 spheroids and migratory cells. (A) Staining pattern for untreated and treated spheroids. **(B)** Staining pattern for untreated and treated migratory cells. Magnification x10.

Supplemental Table 1: Observed staining pattern for Ki67

U87	Core	Migratory	U251 Core	Migratory
Medium	Nuclear	Nuclear	Nuclear	Nuclear
LiCl	Nuclear	Nuclear	Nuclear	Nuclear
BIO	Nuclear	Nuclear	Nuclear	Nuclear

Observed staining pattern for Ki67

Supplemental Table 2: Observed staining pattern for nestin

U87	Core	Migratory	U251 Core	Migratory
Medium	Cyto, PM	Cyto, PM	Cyto, PM	Cyto, PM
LiCl	Cyto, PM	Cyto, PM	Cyto, PM	Cyto, PM
BIO	Cyto, PM	Cyto, PM	Cyto, PM	Cyto, PM

Observed staining pattern for nestin

Supplemental Table 3: Observed staining pattern for cleaved caspase-3

U87	Core	Migratory	U251 Core	Migratory
Medium	Punctate cyto	Cyto, PM	Punctate cyto	A few punctate cyto
LiCl	Punctate cyto	Punctate cyto	Punctate cyto	Punctate cyto
BIO	Cyto	Only a few cyto	Punctate cyto	No labelling

Observed staining pattern for cleaved caspase-3

Supplemental Table 4: Observed staining pattern for SOX-2

U87	Core	Migratory	U251 Core	Migratory
Medium	Cyto	Cyto	Nuclear, diffuse cyto	Cyto
LiCl	Cyto	Cyto	Cyto	Cyto
BIO	Cyto	Cyto	Cyto	Cyto

Observed staining pattern for SOX-2
